# Conductive Hearing Loss Aggravates Memory Decline in Alzheimer Model Mice

**DOI:** 10.3389/fnins.2020.00843

**Published:** 2020-08-13

**Authors:** Jin Su Kim, Hae-June Lee, Seonhwa Lee, Ho Sun Lee, Ye Ji Jeong, Yeonghoon Son, Jung Min Kim, Yong Jin Lee, Min-Hyun Park

**Affiliations:** ^1^Division of RI Application, Korea Institute of Radiological and Medical Sciences, Seoul, South Korea; ^2^Radiological and Medico-Oncological Sciences, University of Science and Technology, Seoul, South Korea; ^3^Division of Radiation Biomedical Research, Korea Institute of Radiological and Medical Sciences, Seoul, South Korea; ^4^Department of Bio-Convergence Engineering, Korea University, Seoul, South Korea; ^5^Department of Otorhinolaryngology, Boramae Medical Center, Seoul Metropolitan Government-Seoul National University, Seoul, South Korea; ^6^Department of Otorhinolaryngology, College of Medicine, Seoul National University, Seoul, South Korea; ^7^National Primate Research Center, Korea Research Institute of Bioscience and Biotechnology (KRIBB), Cheongju, South Korea

**Keywords:** memory impairment, SPM, VBM, Behavioral study, hearing loss, Alzheimer’s disease

## Abstract

The study of cognitive impairment associated with hearing loss has recently garnered considerable interest. Epidemiological data have demonstrated that hearing loss is a risk factor for cognitive decline as a result of aging. However, no previous study has examined the effect of hearing loss in patients with cognitive problems such as Alzheimer’s disease. Therefore, we investigated the effect of conductive hearing loss in an Alzheimer’s mouse model. Positron emission tomography (PET) and magnetic resonance imaging (MRI) were used to evaluate changes in glucose metabolism and gray matter concentrations in the 5xFAD Alzheimer’s Disease (AD) transgenic mouse model with and without conductive hearing loss (HL). Conductive hearing loss was induced using chronic perforation of the tympanic membrane. Behavioral data from the Y-maze and passive avoidance tests revealed greater memory deficits in the AD with HL (AD-HL) group than in the AD group. Following induction of hearing loss, lower cerebral glucose metabolism in the frontal association cortex was observed in the AD-HL group than in the AD group. Although lower glucose metabolism in the hippocampus and cerebellum was found in the AD-HL group than in the AD group at 3 months, the gray matter concentrations in these regions were not significantly different between the groups. Furthermore, the gray matter concentrations in the simple lobule, cingulate/retrosplenial cortex, substantia nigra, retrosigmoid nucleus, medial geniculate nucleus, and anterior pretectal nucleus at 7 months were significantly lower in the AD-HL group than in the AD group. Taken together, these results indicate that even partial hearing loss can aggravate memory impairment in Alzheimer’s disease.

## Introduction

Cognitive impairment associated with hearing loss (HL) has recently attracted considerable interest due to growing evidence suggesting that impaired hearing is a risk factor for cognitive decline (Hardy et al., 2016). Several studies have assessed the relationship between hearing deficits and cognitive impairment. Age-related hearing problems are common among people with dementia and are associated with poor cognitive function and reduced quality of life (Dawes et al., 2018). Furthermore, hearing loss in later life has been associated with the risk of dementia. The impact of the risk factors for dementia may change during a person’s lifespan, and whether or not midlife hearing loss represents a risk factor for dementia remains poorly understood (Osler et al., 2019). Osler et al. have shown that early identification and correction of hearing loss holds promise for the prevention of dementia later in life (Osler et al., 2019). Interventions aimed at improving sensory function may improve the quality of life of patients with dementia (Leroi et al., 2017). Furthermore, middle-aged and old patients with severe or profound hearing impairments also show an increased risk of developing dementia (Kim et al., 2018). However, there are no reports on the effect of hearing loss in patients with cognitive impairment such as Alzheimer’s disease (AD). If hearing loss facilitates cognitive decline in the normal population, then patients with AD and hearing loss may be expected to suffer more in the aspect of cognitive function. Therefore, we investigated the behavior, brain function, and changes in hearing in hearing loss-induced AD model mice using behavioral tests, positron emission tomography (PET), and magnetic resonance (MR) imaging.

Although many reports point to a correlation between dementia and hearing impairment, quantitative and functional studies are challenging because the auditory areas in the brain remain poorly understood and are difficult to assess. Deaf animal models provide us with an opportunity to assess the impact of hearing loss interventions on the development of dementia, as well as the corresponding changes in brain plasticity. We have previously demonstrated cross-modal and compensatory plasticity using PET analysis in an animal model (Park et al., 2010). PET is a promising tool for the assessment of neuronal and cortical plasticity (Lee et al., 2001). The cochlear implant is a surgically implanted neuroprosthetic device that provides a sense of sound to a person with severe to profound sensorineural hearing loss (Balkany et al., 1999; Hodges et al., 1999). PET analyses of brain plasticity have provided quantitative results that support increased metabolic activity in auditory areas following cochlear implantation (Lukaszewicz-Moszynska et al., 2014; Strelnikov et al., 2015; Yoshida et al., 2017). Studies have also assessed verbal working memory in children with cochlear implants (Akcakaya et al., 2019).

The aim of this study was to evaluate the effects of conductive hearing loss on Alzheimer’s disease. We sought to investigate the following questions: (1) Does conductive hearing loss affect memory ability? (2) Does conductive hearing loss lead to functional changes in imaging studies such as PET and MR? Five familial AD mutation (5xFAD) transgenic mice (which are commonly used as an animal model for AD) were used in the current study (Son et al., 2016; O’Leary et al., 2017; Jeon et al., 2018; Moon et al., 2019). Experimental mice were divided into two groups: Alzheimer’s disease with and without hearing loss (AD-HL and AD), and used PET and MR were used to evaluate changes in cerebral glucose metabolism and regional gray matter concentrations after the induction of conductive hearing loss. Memory deficits were assessed using the Y-maze and passive avoidance tests. To the best of our knowledge, our study is the first to investigate the relationship between AD and hearing loss through the evaluation of cerebral glucose metabolism and regional gray matter concentrations using PET and MR image analysis on animal models.

## Materials and Methods

### Animals and Ethics Statement

Five familial AD mutation male mice (2 months of age), which overexpress five familial AD mutations, were used in the current study. These mutations comprise three in human APP (695) with the Swedish (K670N, M671L), Florida (I716V), and London (V7171), and two in human presenilin1, PSEN1 M146L, and PSEN1 L286V. The transgenic mice were purchased from Jackson Laboratory (Bar Horbor, ME, United States). All applicable international, national, and/or institutional guidelines for the care and use of animals were followed. The animal study was approved by the Institutional Animal Care and Use Committee (IACUC) and the Institutional Review Board of the Korea Institute of Radiological and Medical Sciences (KIRAMS 2018-0016, KIRAMS 2015-38), and all experiments were performed in accordance with their guidelines.

### Experimental Design

#### Experimental Group

The animals were divided into two groups: the experimental group (AD-HL; *n* = 10) with induced conductive hearing loss and the control group (AD; *n* = 10) with normal hearing. Both groups underwent measurement of hearing thresholds, behavioral tests, and imaging studies.

#### Hearing Loss

**FIGURE 1:**
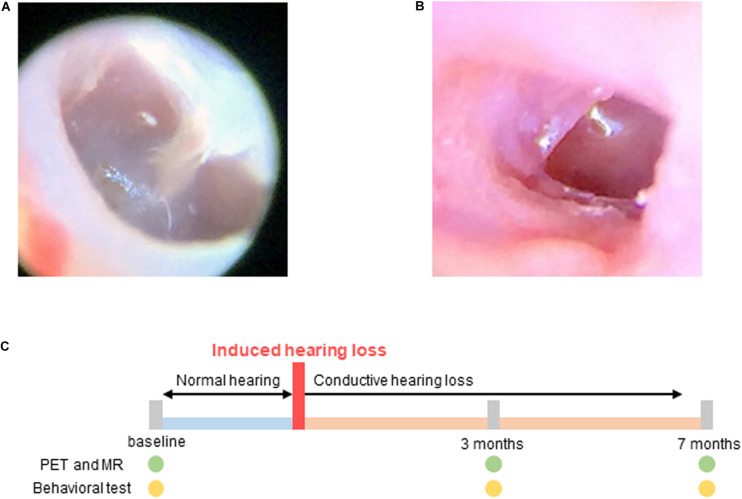
Hearing loss was induced by resection of the tympanic membrane. **(A)** Normal tympanic membrane; **(B)** Resected tympanic membrane; **(C)** Experimental scheme for imaging and behavioral testing. Baseline tests were performed before induced hearing loss. Hearing loss was induced at 0 month, and PET, MR, and behavioral tests were performed 3 and 7 months after hearing loss.

Conductive hearing loss was induced in the experimental group using the following procedure: The mice were anesthetized using intraperitoneal injections of ketamine (100 mg/kg) and xylazine (20 mg/kg). The tympanic membrane was resected using a sharp pick under continuous endoscopic visualization (Liberman et al., 2015). After resection, the movement and balance state of the animals were checked to evaluate inner ear damage. The state of the tympanic membrane was endoscopically examined every week. Resection was repeated when the perforation size was observed to have decreased. The perforation size in the tympanic membrane was maintained until the end of the study (Figure 1).

#### Hearing Evaluation

Hearing levels were assessed using click-evoked ABRs 1 week after surgery, 3 months later, and at the end of the study. Hearing levels were tested by evaluating the auditory brainstem responses (ABRs) to click stimuli (Intelligent Hearing System, Miami, FL, United States). Subdermal needle electrodes were located below both ear and at the vertex. The stimulus rate is 19.3/s using click sound of rarefaction mode. The response was amplified, and band pass filtered (100–3000 Hz), and averaged over stimulation (512 sweeps per stimulation).

### PET Statistical Parametric Mapping (SPM) and MR Voxel-Based Morphometry (VBM) Analyses

PET and MR scans were acquired at baseline and at 3 and 7 months after the induction of hearing loss in order to assess changes in cerebral glucose metabolism and gray matter concentrations (Figure 1C).

Regional cerebral glucose metabolism was measured using the F-18 fluorodeoxyglucose (F-18 FDG) PET scan (Siemens Inveon PET scanner) (Bao et al., 2009). Prior to the PET scan, the mice (*n* = 10 per group, male) underwent an 8-h fasting period, after which they were anesthetized with 2% isoflurane in 100% oxygen (Forane solution; Choongwae Pharma, South Korea). The body temperatures of the mice were maintained at 36°C using heating pads during the course of the scan. Next, 200 μCi of F-18 FDG was injected through the tail vein of the mice. After 30 min of uptake, emission PET data were acquired for 30 min using an energy window of 350–650 keV. The emission list-mode data were sorted into three-dimensional (3D) sinograms and reconstructed using 3D reprojection algorithms without the use of a filter. The size of the image matrix was 256 × 256 × 159, with a pixel size of 0.155 mm × 0.155 mm and a slice thickness of 0.796 mm.

Regional differences between groups were identified using voxel-based statistical analysis performed in SPM 8^1^. Statistical parametric mapping analysis on small animals has been described in our previous study (Kim et al., 2008). Briefly, the brain tissue was extracted from the image and a study-specific mouse brain template was constructed using structural images. Individual PET data were spatially normalized onto the mouse brain template using affine and non-linear transformations. The voxel size of the spatially normalized images was 0.3 mm × 0.3 mm × 0.3 mm. Finally, a Gaussian smoothing kernel with a full-width at half-maximum (FWHM) value of 3 mm was applied to enhance the signal-to-noise ratio. Count normalization was performed. *t*-tests were used to identify regional differences in cerebral glucose metabolism between the groups (*p* < 0.005 or *p* < 0.05, uncorrected).

T2w 3D MR images were acquired using an Agilent 9.4 T MR scanner (United States). An AD quad 70 RF coil was used, and the matrix size was 192 × 192 × 192. The repetition time (RT) was 2500 ms. The effective echo time (TE) was set at 7 ms. The total image acquisition time was 3 h 36 min.

Modulated VBM analysis was performed in SPM eight to compare the regional gray matter concentrations in selected brain regions between the groups (Wilke et al., 2003). Skull stripping was performed using the BrainSuite (version 16) software (Kazemi and Noorizadeh, 2014). Parameters including the brain surface extractor diffusion iterations, diffusion constant, edge constant, and erosion size were adjusted for skull stripping using individual T2w 3D MR data. A predefined gray matter template (matrix size: 512 × 512 × 512; voxel size: 0.04 mm × 0.04 mm × 0.04 mm) created by the Delora research team was used for spatial normalization (Kazemi and Noorizadeh, 2014; Delora et al., 2016). Individual skull-stripped MR data were spatially normalized onto the template and smoothed with a Gaussian smoothing kernel with a full-width at half-maximum (FWHM) value of 2 mm. Paired *t*-tests were used to identify regional differences in gray matter concentrations between the groups (*p* < 0.005, uncorrected).

### Behavioral Analysis

#### Y-Maze Test

The Y-maze test was used to measure spatial working memory and reference memory, which were assessed by recording spontaneous alternation behavior (Kraeuter et al., 2019). Activity was recorded for 8 min and analyzed by a computer program (Viewer3, BIOSERVE, St. Augustin, Germany). Alternation was defined as successive entries into three different arms on overlapping triplet sets. Percentage alternation was calculated as the ratio of actual alternation and possible alternation (defined as the total number of arm entries – 2) × 100, as follows:

%alternation=[(No.ofalternations)/(Totalarmentries-2)]×100.

#### Passive Avoidance Test

The responses to aversive stimuli in the passive avoidance test were used to assess learning and long-term memory (Tucker et al., 1976; Nasri et al., 2012). The setup consisted of two rooms separated by a steel board such that it could automatically be moved up and down to allow movement of the animal from one room to the other. Both the rooms were equipped with a scrambler on the floor, through which electrical stimulation could be delivered to the foot. During the adaptation session (Day 1), the mice were allowed to travel freely between the two rooms for 5 min. On Day 2, the steel board was used to separate the rooms and the mouse was placed in one of the rooms. The room was kept dark for 60 s to allow the mouse to adjust to the darkness, after which the light was turned on and the steel panel was simultaneously removed. As the mouse traveled across to the other room to avoid the bright light, the movement of the mouse triggered the steel panel to shut, and an electrical shock impulse (0.3 mA, 2 s) was transmitted to the grill. After 24 h (Day 3), the mice were subjected to the same trial without the electrical shock stimulus. The time taken by the mice to cross over to the other room was automatically recorded.

### Statistics

Data are presented as mean ± standard error of mean (SEM). For behavioral tests, the differences among the groups were analyzed using the one-way ANOVA test and the difference between two groups were analyzed with Student’s unpaired *t*-test in GraphPad Prism five (GraphPad software, CA, United States). A *P*-value less than 0.05 was considered statistically significant.

## Results

### Measurement of the Level of Hearing Loss

**FIGURE 2:**
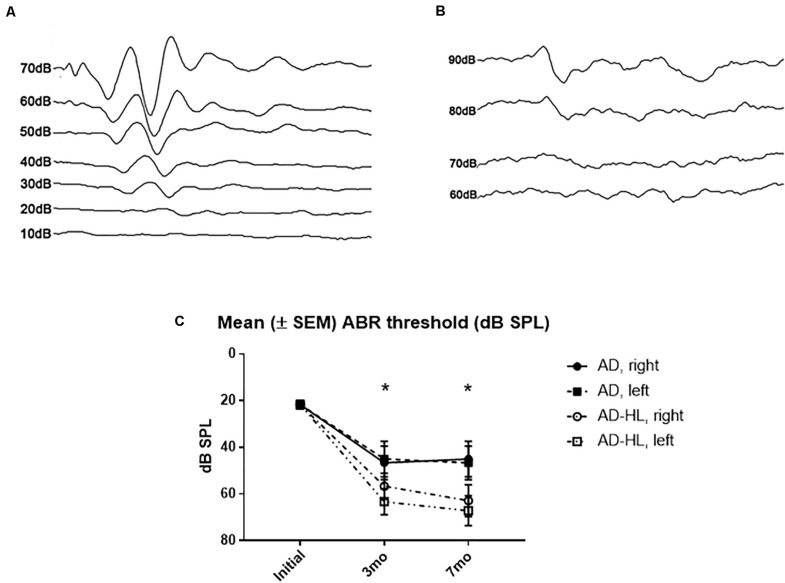
Auditory brainstem responses results in the AD-HL and AD groups. **(A)** Auditory brainstem responses in the AD group revealed a hearing threshold of 20 dB SPL at the initial state. **(B)** Auditory brainstem responses in the AD-HL group revealed a hearing threshold of 65 dB SPL at 3 months. **(C)** Mean (±SEM) ABR threshold (dB SPL) for 3 and 7 months after hearing loss. [ABR: auditory brainstem response, *significant difference between control and hearing loss group at 3 and 7 mo (*P* < 0.05)]. AD-HL, Alzheimer’s disease with hearing loss; AD, Alzheimer’s disease; dB SPL, decibel sound pressure level; SEM, standard error of the means.

The mean hearing level measured using click-evoked ABRs was 20 dB sound pressure level (SPL) at the normal hearing state (Figure 2A). In the control group, the hearing level was increased to 40 dB SPL at 3- and 7-months. In the experimental group, the mean hearing level was 65 dB SPL in both ears after resection of the tympanic membrane (Figure 2B). The final mean hearing levels at 7 months after the induction of hearing loss were 62.9 ± 8 dB SPL on the right side and 67.1 ± 7 dB SPL on the left side (Figure 2C).

### Evidence From PET and MR Imaging

#### Cerebral Glucose Metabolism in the AD Group

**FIGURE 3:**
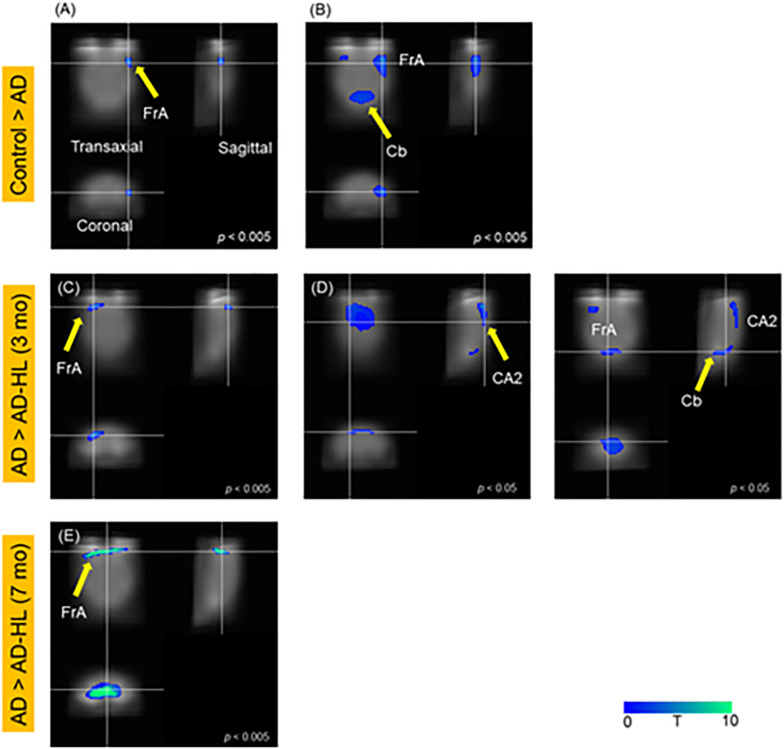
SPM analysis of PET images; **(A,B)** Control vs AD; **(C,D)** AD vs AD-HL (3 mo); **(E)** AD vs AD-HL (7 mo); We found a decrease in the cerebral glucose metabolism in the **(A)** FrA (*p* < 0.005); and **(B)** Cb (*p* < 0.05) at the 3-month timepoint compared to the baseline levels. We found lower cerebral glucose metabolism in the **(C)** FrA (*P* < 0.005), and **(D)** CA2 region of the hippocampus and Cb (*P* < 0.05) in the AD-HL group than in the AD group at the 3-month timepoint after the induction of hearing loss. **(E)** The cerebral glucose metabolism in the FrA was lower in the AD-HL group than in the AD group (*p* < 0.005) at the 7-month time point after the induction of hearing loss. *P-*values were determined using the paired *t*-test. SPM, statistical parametric mapping; AD-HL, Alzheimer’s disease with hearing loss; AD, Alzheimer’s disease; FrA, frontal association cortex; Cb, cerebellum.

SPM analysis of PET scans obtained at baseline and at the 3-month time-point in the AD group revealed decreased cerebral glucose metabolism in the frontal association cortex (FrA; *P* < 0.005; Figure 3A) and the cerebellum (Cb; *P* < 0.05; Figure 3B) at 3 months compared to baseline levels.

#### Comparison Between Cerebral Glucose Metabolism of the AD and AD-HL Groups

Figure 5 illustrates the results of the SPM analysis comparing PET scans of animals in the AD and AD-HL groups at 3 and 7 months after the induction of hearing loss. Cerebral glucose metabolism in the FrA was lower (*P* < 0.005) in the AD-HL group than in the AD model 3 and 7 months after the induction of hearing loss (shown in Figures 3C,E). We also found lower cerebral glucose metabolism in the hippocampus (CA1) and Cb 3 months after the induction of hearing loss (Figure 3D; *P* < 0.05).

#### Cerebral Gray Matter Concentrations in the AD and AD-HL Mice

**FIGURE 4:**
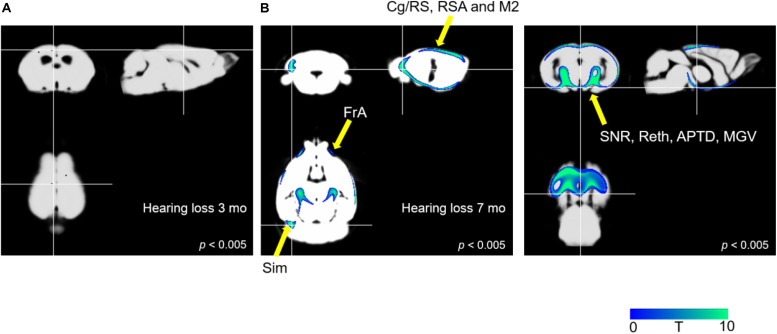
Comparison of VBM results between the AD-HL and AD groups. **(A)** We found no group differences in gray matter concentrations at the 3-month time point after the induction of hearing loss (*P* < 0.005). **(B)** We found a significant decrease in the gray matter concentrations in several regions including the FrA, Sim, RSA, Cg/RS, SNR, REth, MGV, and APTD (*P* < 0.005) at the 7-month time point after the induction of hearing loss. *P-*values were determined using *t*-test. VBM, voxel-based morphometry; AD-HL, Alzheimer’s disease with hearing loss; AD, Alzheimer’s disease; FrA, frontal association cortex; Sim, simple lobule (part of the cerebellum); RSA, retrosplenial agranular cortex; Cg/RS,: cingulate/retrosplenial cortex; SNR, substantia nigra; REth, retroethmoid nucleus; MGV, medial geniculate nucleus; APTD, anterior pretectal nucleus.

Figure 4 illustrates the results of the optimized VBM analysis comparing the MR images of mice in the AD and AD-HL groups. We found no differences in the gray matter concentrations of the two groups at the 3-month time-point after the induction of hearing loss (*P* < 0.005). However, at the 7-month time-point after the induction of hearing loss, the AD-HL group showed lower gray matter concentrations in large areas of the brain including the FrA, simple lobule (Sim, part of the cerebellum), RSA (retrosplenial agranular cortex), cingulate/retrosplenial cortex (Cg/RS), substantia nigra (SNR), retroethmoid nucleus (REth), medial geniculate nucleus (MGV), and anterior pretectal nucleus (APTD) than the AD group (*P* < 0.005).

### Behavioral Analysis

**FIGURE 5:**
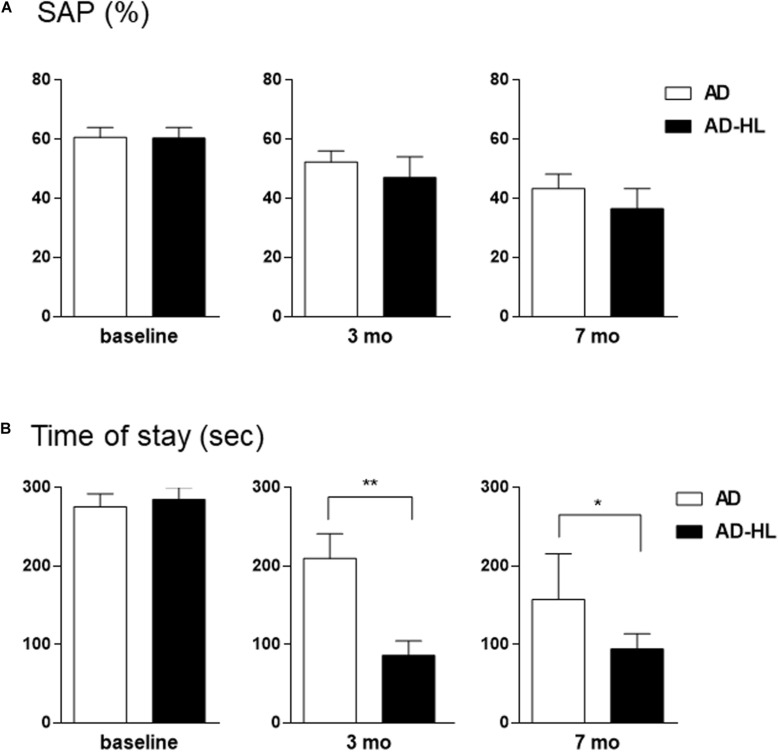
Comparison of behavioral changes between the AD-HL and AD groups. **(A)** In the Y-maze, we found no significant group differences in the percentage of spontaneous alternation at any of the tested time-points. However, the AD group tended to exhibit higher spatial memory than the AD-HL group at the 3- and 7-month time-points. **(B)** In the passive avoidance memory test, compared to the AD group, the AD-HL group exhibited significantly higher latency time at the 3- and 7-month time-points, indicating higher memory impairment. Data are represented as mean ± SEM. *P < 0.05, **P < 0.01 (vs AD group). *P*-values were calculated using *t*-test. AD-HL, Alzheimer’s disease with hearing loss; AD, Alzheimer’s disease; SEM, standard error of the means; SAP, spontaneous alternation percentage.

The AD-HL and AD groups underwent behavioral assessment prior to the induction of conductive hearing loss (baseline) when they were 6 weeks old, and at the 3- and 7-month time-points after induction of hearing loss (in the AD-HL group). None of the animals showed balance or movement problems after resection of tympanic membrane. We assessed the effect of hearing loss on spatial memory using the Y-maze test. The AD group showed an age-dependent decline in working memory. We found no significant difference between the AD and AD-HL groups in the spontaneous alternation as evaluated by the Y-maze (Figure 5A) at the 3- and 7-month time-points after hearing loss. However, the AD group tended to show higher spatial memory compared to the AD-HL group.

The passive avoidance test was performed to examine the hearing loss-induced memory impairment in the AD-HL group. We found no significant difference in cognitive performance in the passive avoidance test between the two groups prior to the induction of hearing loss. The AD-HL group exhibited significantly higher memory impairment 24 h after the training sessions with the electric shock stimulus than the AD group at the 3-month (mean latency: 210 s in AD vs 87 s in AD-HL, *p* = 0.0037) and 7-month (mean latency: 157 s in AD vs 95 s in AD-HL, *p* = 0.046) time-points following hearing loss induction (Figure 5B).

## Discussion

Several meta-analyses, including a meta-analysis of a prospective cohort study, have suggested that hearing impairment significantly increases the risk of developing cognitive disorders (Zheng et al., 2017). The importance of evaluating hearing levels and administering appropriate rehabilitation treatments as part of the cognitive assessment and management plan in individuals with cognitive disorders has also been emphasized (Nirmalasari et al., 2017). Further, age-related hearing loss has been considered to be a possible biomarker and modifiable risk factor for cognitive decline, cognitive impairment, and dementia (Loughrey et al., 2018).

Although meta-analyses and cohort studies have indicated a relationship between hearing loss (hearing impairment or deafness) and cognitive impairment, PET or MR image analyses or behavioral studies have not been employed to further characterize the relationship until now. Imaging studies using PET have provided a translational platform for clinical use and successful clinical proof-of-concept testing (Platt et al., 2011; Hargreaves and Rabiner, 2014). FDG-PET imaging has been used to visualize the distribution of neural damage or synaptic dysfunction; to identify distinct phenotypes of neurodegenerative disorders such as AD (McKhann et al., 2011), and to identify changes in cerebral glucose metabolism after the onset of deafness, or in brain plasticity after cochlear implantation (Okuda et al., 2013; Strelnikov et al., 2015; Suh et al., 2015; Yoshida et al., 2017). In the present study, we constructed an AD-HL animal model. This 5xFAD mouse showed peripheral hearing loss with aging (O’Leary et al., 2017). At 4-months-old, the hearing level was normal, but at 14-months-old, the hearing level was much lower than that of the wild type mouse; this has been shown to be an aggravating factor for cognitive decline. Another study was used an animal model of amyloid-β infusion to intracerebroventricular space in order to generate a suitable model for AD; in this study, deafness was induced using cochlear ablation Cognitive behavioral tests were subsequently performed, and amyloid-β infused group showed poor performance and greater loss of synapses in the hippocampus (Chang et al., 2019).

In the current study, we assessed cerebral glucose metabolism, regional gray matter concentrations changes using PET, MR imaging, and behavioral tests. We found reduced glucose metabolism in the FrA (*P* < 0.005), hippocampus, and Cb (*P* < 0.05) in the AD-HL group 3 months after the induction of hearing loss. Interestingly, this functional change was not accompanied by changes in regional gray matter concentrations at the corresponding sites. Memory decline was confirmed using the passive avoidance test. Lower cerebral glucose metabolism levels in the FrA and hippocampus related to memory deficits have also been found in our previous PET study (Park et al., 2013; Lim et al., 2016; Ye et al., 2016). Furthermore, other groups have reported cognitive decline-associated decreases in cerebral glucose metabolism in the Cb (Schreck et al., 2018; Liang and Carlson, 2019; Nishida et al., 2019). In the current study, the decrease in the cerebral glucose metabolism in the FrA persisted at the 7-month time-point. Furthermore, VBM analysis also revealed decreases in gray matter concentrations in the Sim, RSA, and Cg/RS in the AD-HL group. The Cg/RS and RSA are known to be the key brain areas involved in memory, emotion, and attention functions (Zgraggen et al., 2012; Milczarek et al., 2018).

However, we noted a discrepancy in the results from PET and MR image analyses. Although we found group differences in the regional gray matter concentrations in several areas of the brain at the 7-month time point, SPM analysis of PET imaging data did not reveal differences in glucose metabolism at the corresponding sites, except in the case of the FrA. This discrepancy could be explained by brain plasticity. Although the glucose metabolism decreased in the hippocampus and Cb 3 months after the induction of hearing loss, the metabolism in these regions recovered by 7 months, possibly due to brain plasticity. This type of recovery has been previously reported both in humans and cats (Lee et al., 2001; Park et al., 2010). In previous studies, the primary auditory cortex showed hypometabolism after deafness, and then, the hypometabolic area was normalized further after even the ear was still deaf. They explained this phenomenon as an evidence of cross-modal plasticity. Therefore, in our study, CA2 area showed hypometabolism at 3 months after hearing loss; however, the change was normalized at 7 months after hearing loss, even the hearing loss and behavioral deficit were still remained. In addition, the results of the VBM analysis suggest differences in gray matter concentration in several areas of the brain, including the FrA.

Our findings suggest that the decrease in cerebral glucose metabolism in the hippocampus is correlated with memory deficits, such as those affecting long-term memory (Sawangjit et al., 2018). The results of the passive avoidance test support the development of memory deficits induced by hearing loss, especially in terms of long-term memory. Additionally, we found no changes in regional gray matter concentrations in the whole brain at the 3-month time-point, which implies that there were changes only in the glucose metabolism at this time. However, we found significant changes in the gray matter concentrations in memory and motor function-related brain areas at 7 months after the induction of hearing loss. SPM analysis of PET images at 7 months revealed a recovery in the impaired glucose metabolism in the Cb at this time point.

Interestingly, we did not find significant changes in glucose metabolism or gray matter concentration in the auditory cortex at the 3- or 7-month time-points after the onset of hearing loss. We believe this may be because our mouse model with hearing loss did not constitute complete deafness. In our conductive hearing loss model, the mice could still hear loud sounds. As the auditory system was still functional, loud sounds could evoke activation of the auditory system. However, we found lower cerebral glucose metabolism in the FrA in the AD-HL group than in the AD group. The FrA is composed of the prefrontal cortex and motor function-related areas of the brain excluding the primary motor cortex (Takada, 2016). Neurophysiology and neuropsychology studies have established that the dorsolateral prefrontal cortex is associated with working memory, while the ventral frontal lobe is associated with auditory and audiovisual working memory (Plakke et al., 2015). Our PET findings revealed decreased glucose metabolism in the dorsolateral prefrontal cortex at the 3-month time-point after induction of hearing loss in the AD-HL mice. However, by the 7-month time-point, the glucose metabolism in the ventral prefrontal cortex, as well as in the dorsolateral prefrontal cortex regions was affected. This finding suggests that auditory areas including the ventral prefrontal cortex were affected despite the fact that we did not induce complete hearing loss.

## Conclusion

Our findings reveal memory impairment after hearing loss in the AD mice as evidenced by PET and MR imaging findings. The results from our behavioral tests also support an association between memory impairment and hearing loss. Together, these results suggest that hearing loss may aggravate memory decline in an animal model of AD.

In sum, we constructed an AD-HL animal model and assessed changes in cerebral glucose metabolism and gray matter concentrations following hearing loss. Our results provide experimental evidence to suggest that even partial hearing loss can aggravate memory impairment in AD. In the future, this model can also be used to identify the onset of memory deficit and brain plasticity following the onset of hearing loss.

## Data Availability Statement

The raw data supporting the conclusions of this article will be made available by the authors, without undue reservation.

## Ethics Statement

The animal study was approved by the Institutional Animal Care and Use Committee (IACUC) and the Institutional Review Board of the Korea Institute of Radiological and Medical Sciences (KIRAMS 2018-0016, and KIRAMS 2015-38), and all experiments were performed in accordance with their guidelines.

## Author Contributions

JK and M-HP designed the study, analyzed and interpreted the results, and wrote the manuscript. H-JL, SL, HL, YJ, and YS performed experiments, analyzed data, and wrote the manuscript. YL discussed the results and edited the manuscript. All authors reviewed and approved the final version of the manuscript.

## Conflict of Interest

The authors declare that the research was conducted in the absence of any commercial or financial relationships that could be construed as a potential conflict of interest.

## Footnotes

1 http://www.fil.ion.ucl.ac.uk/spm

## References

[B1] Akcakaya H. Dogan M. Gurkan S. Kocak O. Yucel E. (2019). Early cochlear implantation: verbal working memory, vocabulary, speech intelligibility and participant variables. *Cochlear Implants Int.* 20 62–73. 10.1080/14670100.2019.1565077 30621508

[B2] Balkany T. J. Hodges A. V. Gomez-Marin O. Bird P. A. Dolan-Ash S. Butts S. (1999). Cochlear reimplantation. *Laryngoscope* 109 351–355. 10089956 10.1097/00005537-199903000-00002

[B3] Bao Q. Newport D. Chen M. Stout D. B. Chatziioannou A. F. (2009). Performance evaluation of the inveon dedicated PET preclinical tomograph based on the NEMA NU-4 standards. *J. Nucl. Med.* 50 401–408. 10.2967/jnumed.108.056374 19223424 PMC2803022

[B4] Chang M. Kim H. J. Mook-Jung I. Oh S. H. (2019). Hearing loss as a risk factor for cognitive impairment and loss of synapses in the hippocampus. *Behav. Brain Res.* 372:112069. 10.1016/j.bbr.2019.112069 31271817

[B5] Dawes P. Wolski L. Himmelsbach I. Regan J. Leroi I. (2018). Interventions for hearing and vision impairment to improve outcomes for people with dementia: a scoping review. *Int. Psychogeriatr.* 31 203–221. 10.1017/S1041610218000728 30244688

[B6] Delora A. Gonzales A. Medina C. S. Mitchell A. Mohed A. F. Jacobs R. E. (2016). A simple rapid process for semi-automated brain extraction from magnetic resonance images of the whole mouse head. *J. Neurosci. Methods* 257 185–193. 10.1016/j.jneumeth.2015.09.031 26455644 PMC4910826

[B7] Hardy C. J. Marshall C. R. Golden H. L. Clark C. N. Mummery C. J. Griffiths T. D. (2016). Hearing and dementia. *J. Neurol.* 263 2339–2354. 10.1007/s00415-016-8208-y 27372450 PMC5065893

[B8] Hargreaves R. J. Rabiner E. A. (2014). Translational PET imaging research. *Neurobiol. Dis.* 61 32–38. 10.1016/j.nbd.2013.08.017 24055214

[B9] Hodges A. V. Villasuso E. Balkany T. Bird P. A. Butts S. Lee D. (1999). Hearing results with deep insertion of cochlear implant electrodes. *Am. J. Otol.* 20 53–55. 9918173

[B10] Jeon S. G. Kang M. Kim Y. S. Kim D. H. Nam D. W. Song E. J. (2018). Intrahippocampal injection of a lentiviral vector expressing neurogranin enhances cognitive function in 5XFAD mice. *Exp. Mol. Med.* 50:e461. 10.1038/emm.2017.302 29568074 PMC5898899

[B11] Kazemi K. Noorizadeh N. (2014). Quantitative comparison of SPM, FSL, and brainsuite for brain MR image segmentation. *J. Biomed. Phys. Eng.* 8 13–26. PMC4258855 25505764

[B12] Kim J. S. Lee J. S. Park M. H. Kang H. Lee J. J. Lee H. J. (2008). Assessment of cerebral glucose metabolism in cat deafness model: strategies for improving the voxel-based statistical analysis for animal PET studies. *Mol. Imaging Biol.* 10 154–161. 10.1007/s11307-008-0140-9 18425556

[B13] Kim S. Y. Lim J. S. Kong I. G. Choi H. G. (2018). Hearing impairment and the risk of neurodegenerative dementia: a longitudinal follow-up study using a national sample cohort. *Sci. Rep.* 8:15266. 10.1038/s41598-018-33325-x 30323320 PMC6189102

[B14] Kraeuter A. K. Guest P. C. Sarnyai Z. (2019). The Y-Maze for assessment of spatial working and reference memory in mice. *Methods Mol. Biol.* 1916 105–111. 10.1007/978-1-4939-8994-2_10 30535688

[B15] Lee D. S. Lee J. S. Oh S. H. Kim S. K. Kim J. W. Chung J. K. (2001). Cross-modal plasticity and cochlear implants. *Nature* 409 149–150. 10.1038/35051653 11196628

[B16] Leroi I. Pye A. Armitage C. J. Charalambous A. P. Constantinidou F. Helmer C. (2017). Research protocol for a complex intervention to support hearing and vision function to improve the lives of people with dementia. *Pilot Feasibility Stud.* 3:38. 10.1186/s40814-017-0176-1 28912959 PMC5594580

[B17] Liang K. J. Carlson E. S. (2019). Resistance, vulnerability and resilience: a review of the cognitive cerebellum in aging and neurodegenerative diseases. *Neurobiol. Learn. Mem.* 170:106981. 10.1016/j.nlm.2019.01.004 30630042 PMC6612482

[B18] Liberman M. C. Liberman L. D. Maison S. F. (2015). Chronic conductive hearing loss leads to cochlear degeneration. *PLoS One* 10:e0142341. 10.1371/journal.pone.0142341 26580411 PMC4651495

[B19] Lim I. Joung H. Y. Yu A. R. Shim I. Kim J. S. (2016). PET evidence of the effect of donepezil on cognitive performance in an animal model of chemobrain. *Biomed. Res. Int.* 2016:6945415. 10.1155/2016/6945415 27556039 PMC4983340

[B20] Loughrey D. G. Kelly M. E. Kelley G. A. Brennan S. Lawlor B. A. (2018). Association of age-related hearing loss with cognitive function, cognitive impairment, and dementia: a systematic review and meta-analysis. *JAMA Otolaryngol. Head Neck Surg.* 144 115–126. 10.1001/jamaoto.2017.2513 29222544 PMC5824986

[B21] Lukaszewicz-Moszynska Z. Lachowska M. Niemczyk K. (2014). Auditory cortical activation and plasticity after cochlear implantation measured by PET using fluorodeoxyglucose. *Funct. Neurol.* 29 121–125. 25306122 PMC4198160

[B22] McKhann G. M. Knopman K. D. Chertkow H. Hyman B. T. Jack C. R. Jr. Kawas C. H. (2011). The diagnosis of dementia due to Alzheimer’s disease: recommendations from the national institute on aging-Alzheimer’s association workgroups on diagnostic guidelines for Alzheimer’s disease. *Alzheimers Dement.* 7 263–269. 21514250 10.1016/j.jalz.2011.03.005 PMC3312024

[B23] Milczarek M. M. Vann S. D. Sengpiel F. (2018). Spatial memory engram in the mouse retrosplenial cortex. *Curr. Biol.* 28 1975–1980.e6. 10.1016/j.cub.2018.05.002 29887312 PMC6013279

[B24] Moon M. Jung E. S. Jeon S. G. Cha M. Y. Jang Y. Kim W. (2019). Nurr1 (NR4A2) regulates Alzheimer’s disease-related pathogenesis and cognitive function in the 5XFAD mouse model. *Aging Cell* 18:e12866. 10.1111/acel.12866 30515963 PMC6351845

[B25] Nasri S. Roghani M. Baluchnejadmojarad T. Balvardi M. Rabani T. (2012). Chronic cyanidin-3-glucoside administration improves short-term spatial recognition memory but not passive avoidance learning and memory in streptozotocin-diabetic rats. *Phytother. Res.* 26 1205–1210. 10.1002/ptr.3702 22228592

[B26] Nirmalasari O. Mamo S. K. Nieman C. L. Simpson A. Zimmerman J. Nowrangi M. A. (2017). Age-related hearing loss in older adults with cognitive impairment. *Int. Psychogeriatr.* 29 115–121. 10.1017/S1041610216001459 27655111 PMC6296752

[B27] Nishida Y. Hizume M. Fumimura Y. Ichikawa T. (2019). Cerebellar cognitive affective syndrome improved by donepezil. *Intern. Med.* 58 1003–1006. 10.2169/internalmedicine.1206-18 30568118 PMC6478979

[B28] Okuda T. Nagamachi S. Ushisako Y. Tono T. (2013). Glucose metabolism in the primary auditory cortex of postlingually deaf patients: an FDG-PET study. *ORL J. Otorhinolaryngol. Relat. Spec.* 75 342–349. 10.1159/000357474 24435067

[B29] O’Leary T. P. Shin S. Fertan E. Dingle R. N. Almuklass A. Gunn R. K. (2017). Reduced acoustic startle response and peripheral hearing loss in the 5xFAD mouse model of Alzheimer’s disease. *Genes Brain Behav.* 16 554–563. 10.1111/gbb.12370 28133939

[B30] Osler M. Christensen G. T. Mortensen E. L. Christensen K. Garde E. Rozing M. P. (2019). Hearing loss, cognitive ability, and dementia in men age 19-78 years. *Eur. J. Epidemiol.* 34 125–130. 10.1007/s10654-018-0452-2 30306425

[B31] Park H. J. Shim H. S. Kim K. S. Han J. J. Kim J. S. Ram Yu A. (2013). Enhanced learning and memory of normal young rats by repeated oral administration of Krill Phosphatidylserine. *Nutr. Neurosci.* 16 47–53. 10.1179/1476830512Y.0000000029 22889566

[B32] Park M. H. Lee H. J. Kim J. S. Lee J. S. Lee D. S. Oh S. H. (2010). Cross-modal and compensatory plasticity in adult deafened cats: a longitudinal PET study. *Brain Res.* 1354 85–90. 20692241 10.1016/j.brainres.2010.07.105

[B33] Plakke B. Hwang J. Romanski L. M. (2015). Inactivation of primate prefrontal cortex impairs auditory and audiovisual working memory. *J. Neurosci.* 35 9666–9675. 10.1523/JNEUROSCI.1218-15.2015 26134649 PMC4571503

[B34] Platt B. Welch A. Riedel G. (2011). FDG-PET imaging, EEG and sleep phenotypes as translational biomarkers for research in Alzheimer’s disease. *Biochem. Soc. Trans.* 39 874–880. 10.1042/BST0390874 21787316

[B35] Sawangjit A. Oyanedel C. N. Niethard N. Salazar C. Born J. Inostroza M. (2018). The hippocampus is crucial for forming non-hippocampal long-term memory during sleep. *Nature* 564 109–113. 10.1038/s41586-018-0716-8 30429612

[B36] Schreck L. Ryan S. Monaghan P. (2018). Cerebellum and cognition in multiple sclerosis. *J. Neurophysiol.* 120 2707–2709. 10.1152/jn.00245.2018 30110238

[B37] Son S. M. Shin H. J. Byun J. Kook S. Y. Moon M. Chang Y. J. (2016). Metformin facilitates amyloid-beta generation by beta- and gamma-secretases via autophagy activation. *J. Alzheimers Dis.* 51 1197–1208. 10.3233/JAD-151200 26967226

[B38] Strelnikov K. Marx M. Lagleyre S. Fraysse B. Deguine O. Barone P. (2015). PET-imaging of brain plasticity after cochlear implantation. *Hear. Res.* 322 180–187. 10.1016/j.heares.2014.10.001 25448166

[B39] Suh M. W. Park K. T. Lee H. J. Lee J. H. Chang S. O. Oh S. H. (2015). Factors contributing to speech performance in elderly cochlear implanted patients: an FDG-PET study: a preliminary study. *J. Int. Adv. Otol.* 11 98–103. 10.5152/iao.2015.424 26380996

[B40] Takada M. (2016). [Neuroanatomy of frontal association cortex]. *Brain Nerve* 68 1253–1261. 10.11477/mf.1416200588 27852016

[B41] Tucker A. R. Gibbs M. E. Stanes M. D. (1976). Cycloheximide and passive avoidance memory in mice: time-response, dose-response and short-term memory. *Pharmacol. Biochem. Behav.* 4 441–446. 935215 10.1016/0091-3057(76)90061-7

[B42] Wilke M. Kassubek J. Ziyeh S. Schulze-Bonhage A. Huppertz H. J. (2003). Automated detection of gray matter malformations using optimized voxel-based morphometry: a systematic approach. *Neuroimage* 20 330–343. 14527593 10.1016/s1053-8119(03)00296-9

[B43] Ye M. Chung H. S. An Y. H. Lim S. J. Choi W. Yu A. R. (2016). Standardized herbal formula PM012 decreases cognitive impairment and promotes neurogenesis in the 3xTg AD mouse model of Alzheimer’s disease. *Mol. Neurobiol.* 53 5401–5412. 10.1007/s12035-015-9458-x 26446019

[B44] Yoshida H. Takahashi H. Kanda Y. Chiba K. (2017). PET-CT observations of cortical activity in pre-lingually deaf adolescent and adult patients with cochlear implantation. *Acta Otolaryngol.* 137 464–470. 10.1080/00016489.2016.1253868 27841068

[B45] Zgraggen E. Boitard M. Roman I. Kanemitsu M. Potter G. Salmon P. (2012). Early postnatal migration and development of layer II pyramidal neurons in the rodent cingulate/retrosplenial cortex. *Cereb. Cortex* 22 144–157. 10.1093/cercor/bhr097 21625013

[B46] Zheng Y. Fan S. Liao W. Fang W. Xiao S. Liu J. (2017). Hearing impairment and risk of Alzheimer’s disease: a meta-analysis of prospective cohort studies. *Neurol. Sci.* 38 233–239. 10.1007/s10072-016-2779-3 27896493

